# “Smelling and Tasting” Parkinson's Disease: Using Senses to Improve the Knowledge of the Disease

**DOI:** 10.3389/fnagi.2020.00043

**Published:** 2020-02-25

**Authors:** Valentina Oppo, Marta Melis, Melania Melis, Iole Tomassini Barbarossa, Giovanni Cossu

**Affiliations:** ^1^Department of Neuroscience, Brotzu Hospital, Cagliari, Italy; ^2^Department of Neurology, Azienda Ospedaliero Universitaria, University of Cagliari, Cagliari, Italy; ^3^Department of Biomedical Sciences, University of Cagliari, Cagliari, Italy

**Keywords:** smell, taste, Parkinson, microbiota, mild cognitive impairment

## Abstract

Among non-motor manifestations of Parkinson's Disease (PD), peripheral, sensory symptoms are particularly relevant. Smell dysfunction starts very early and frequently precedes the PD motor symptoms by years (being often a cue to the diagnosis). Moreover, olfactory system could be, together with gut, one of those peripheral sites where PD pathology first develops. Unlike smell loss, the relationship between PD and taste impairment is far less established. It can start early in the course of the disease but more frequently appears in advanced stages, in parallel with the advent of MCI, likely reflecting cortical involvement. Among PD patients has been demonstrated an increase in the frequency of the non-tasters for PROP (prototypical gustatory stimulus, 6- n-propylthiouracil), a genetically determined bitter taste which is mediated by TAS2RS38 receptor, and a significant increase of the recessive non-testing variant of this receptor. TAS2R38 receptors are expressed also in other tissues, such as in the epithelia of the gut and nasal cavities, where they can influence epithelial immunity ad its interaction with microbiota. Those pieces of evidence suggest that not only systematic assessment of taste and smell can be of a remarkable help for clinicians in the early diagnosis, but also that understanding the mechanisms of sensory involvement in PD could increase the knowledge of the pathophysiology of the disease.

## Introduction

The clinical diagnosis of Parkinson's disease (PD) is straightforward when the visible motor signs are present, yet the sensory symptoms may precede or go together with the development of parkinsonism and are an integral part of neurodegeneration. Their systematic assessment can be of remarkable help for clinicians in the early diagnosis or for a better understanding of the disorder pathophysiology. Smell has been extensively studied in PD and prospective studies clearly demonstrated that olfactory impairment is associated with a higher risk in the disease development (Haehner et al., 2007).

Taste impairment, i.e., defective sweet, sour, bitter and salty perception, has also been demonstrated in PD (Cecchini et al., 2014), though it has received much less scientific attention than smell, probably also because the impact of these symptoms on quality of life was considered modest. In this review, we assess the current knowledge about smell and taste disorders in PD, seeking to understand their pathologic underpinnings.

## Smell

### Physiology

The olfactory mucosa represents a proportion (~1.25%) of the nasal mucosa in humans (Godoy et al., 2015). The about 10 million of dendrites of olfactory receptor neurons in the olfactory bulb project to the mucosa. Olfactory sensory neurons send their axons to olfactory glomeruli. Here they make synapses with mitral and tufted cells that have varies projections, through the olfactory tract, to primary olfactory cortex, amygdala, entorhinal cortex, and many cortical projections (Patel and Pinto, 2014). In humans there are ~350 functional odorant receptor genes each coding to the specific receptor sensing its own individual subset of chemicals or substances and leading to the complex mechanism of smell identification (Garcia-Esparcia et al., 2013).

### Smell and PD

It is well-known that olfactory impairment is a common finding in Parkinson's Disease (PD) and it occurs generally early in the course of the disease. Reported rates of smell impairment in PD patients range from 75 to 95% (Haehner et al., 2009, 2019; Doty, 2012; Haugen et al., 2016) in comparison with 25% in normal population (Murphy et al., 2002). Olfactory impairment can be associated with a 10% increased risk of future PD (Ponsen et al., 2004) and, in healthy subjects, hyposmia together with impaired DAT scan is highly predictive of PD (Jennings et al., 2017). Moreover, smell impairment is reliable in differentiating early-stage PD from age-matched controls (Doty et al., 1995) and the olfaction assessment can be useful to differentiate PD from other conditions related to PD, but without a strong association with smell impairment: Essential Tremor (ET) (Busenbark et al., 1992) and atypical parkinsonian disorders like Multiple System Atrophy (MSA), Progressive Nuclear Palsy (PSP), and Corticobasal Degeneration (CBD) (Doty et al., 1993; Wenning et al., 1995; Krismer et al., 2017). Olfactory loss is therefore considered a supportive criterion for PD diagnosis (Postuma et al., 2015).

Differently from general population, where smoking is related with impairment of olfactory function (Katotomichelakis et al., 2007), patients smokers among PD patients have less decline in their olfactory function when compared to those who do not smoke (Sharer et al., 2015). Among PD patients anosmia has been associated with worse performance on cognitive tests and it may be a predictor of emergent PD-related dementia (Baba et al., 2012). Interestingly, smell impairment in PD has been linked to impairment of cholinergic transmission. In fact, cholinergic denervation of the limbic archicortex (assessed through [(11)C]methyl-4-piperidinyl propionate acetylcholinesterase brain positron emission tomography) was found to be a more robust determinant of hyposmia than nigrostriatal dopaminergic denervation in patients with moderately severe PD without dementia (Bohnen et al., 2010). These data are in line with the observation that hyposmia does not improve with levodopa (Tarakad and Jankovic, 2017), while some evidences suggest that rasagiline is associated with significantly better odor discrimination abilities in early-PD patients (Haehner et al., 2015).

Smell impairment is more frequent in male patients (Picillo et al., 2013) and is more severe in those subtypes of PD associated with postural instability and gait disorder (PIGD) (Stern et al., 1994), and has been found to be more prevalent in rigid-akinetic PD than in tremor-dominant type (Iijima et al., 2011). So, different degrees of smell disorder could be related to a different extent of neurodegeneration of the nigrostriatal dopaminergic neurons, implying a worse prognosis in patients with more marked hyposmia (Iijima et al., 2011). According to this observation, worse olfactory performance was recently found in patients with higher Hoehn and Yahr stage (Jalali et al., 2019).

Most patients have asymptomatic smell loss that does not progress with time, because the maximum impairment appears to be reached early on (Katzenschlager et al., 2004).

Smell impairment has a not negligible impact on quality of life. In the general population, hyposmic older adults are more likely to experience depressive symptoms, due to impairment of food and drink enjoyment and socializing (Gopinath et al., 2011).

Nonetheless, smell impairment is often poorly perceived: PD patients are often unaware of it (White et al., 2016), this possibly leading to its under recognition.

As a non-dopaminergic PD manifestation, there are no effective pharmacological treatments for smell impairment, although some data point to a small benefit of rasagiline on smell impairment in early PD (Haehner et al., 2015). Among non-pharmacological treatments, olfactory training has been proven capable to improve smell sensitivity in a small cohort of PD patients (Haehner et al., 2013).

Smell impairment often predates onset of motor features by at least 4 years (Ross et al., 2008), being probably the earliest sign of central nervous system pathology in PD. Parkinsonism is in fact associated with abnormalities in several central regions involved in odor perception (Tarakad and Jankovic, 2017): (1) olfactory bulb volume is decreased in PD patients (Pearce et al., 1995; Wang et al., 2011); (2) Increased diffusivity on MRI diffusion weighted imaging was found in the region of both olfactory tracts in patients with PD compared to controls (Scherfler et al., 2006); (3) α-synuclein deposition in the olfactory tract and anterior olfactory nucleus was demonstrated on pathological examination of patients even in the earliest stages of PD (Braak et al., 2003a), and also in the olfactory bulb (Pearce et al., 1995), while Lewy neurites were identified in the olfactory mucosa (Funabe et al., 2013). Individual variations of olfactory performance of PD patients have been associated with gender (Doty et al., 1995; Morley et al., 2018; Melis et al., 2019) and with a polymorphism of genes coding for odorant-binding protein OBPIIa (Melis et al., 2019), as already shown in healthy subjects (Tepper et al., 2017; Tomassini Barbarossa et al., 2017; Sollai et al., 2019). The genotype AA in the locus in OBPIIa seems to preserve the olfactory function in PD women (Melis et al., 2019).

The main aspects associated with smell impairment in PD are summarized in Table 1.

**Table 1:** Summarizes the main PD clinical and demographic features associated with smell and taste impairment.

**Factors associated with sense impairment in PD**	**Sense**	
	**Smell**	**Taste**
Gender	Male	Male
Age	Older age	Older Age
Disease fenotype	Rigid-Akinetic type, PIGD type	No difference
Associated features	Cognitive impairmentgayu Higher H&Y stage	Cognitive impairment
Appearance at the stage of illness	Early/Prodromic	Intermediate/Late
Smoking habits	Non smokers	Not investigated
Prevalence	75–90%	≈10%
Treatment	Olfactory training	No specific treatment

### Role of Smell in the PD Pathological Process and Relationship With the Microbiota

According to Braak's model (Hawkes et al., 2007), the pathological process of PD starts at the same time in two sites, the olfactory bulb/anterior olfactory nucleus, and the enteric nerve cell plexuses. This pathogenic explanation is known as the “dual-hit” hypothesis. Constipation is actually another well-characterized, early prodromal manifestation of PD.

The alfa-synuclein pathology spreads in a caudal-rostral fashion from the lower brainstem through mid- and forebrain, up to the cerebral cortex in the final stages. Always according to this hypothesis (Braak et al., 2003b), a yet unknown pathogen could be responsible for this stereotypical sequential damage of the nervous system areas, accessing the Central Nervous System (CNS) via the olfactory bulb and the myenteric plexus of the enteric nervous system (ENS). Those two sites are especially vulnerable due to their lack of a blood brain barrier (BBB), that surrounds the CNS (Braak et al., 2003b; Hawkes et al., 2007; Mori, 2015; Sampson et al., 2016). This alleged pathogen could trigger neurodegeneration through a prion-like diffusion of misfolded proteins along neural pathways, or by provoking neuroinflammation leading to degeneration (Bell et al., 2019).

A growing body of evidence indicates a key role in this process of components of humans' tissue-resident micro-organisms, the microbiota, 10–100 trillion symbiotic microbial cells harbored by each person, primarily bacteria in the gut (Ursell et al., 2012). While differences in composition of gut microbiota between PD patients and healthy controls are well-established (Keshavarzian et al., 2015; Scheperjans et al., 2015) and could influence the phenotypical expression of the disease (Scheperjans et al., 2015), it is less clear whether such differences exist for nasal microbiota composition. Pereira et al. did not find any significant differences in nasal microbiota composition of healthy controls and PD patients (Pereira et al., 2017). They were though, as they stated, unable to sample the olfactory mucosa nearest to the olfactory bulb. It has been demonstrated that spatial variability of microbial communities exists in the nasal cavity (Yan et al., 2013). Another recent study did not find any consistent difference in the nasal microbiota composition between PD patients and healthy controls (Heintz-Buschart et al., 2018). Moreover, a high interindividual variability was noted for the nasal microbiota, with sex as the strongest grouping factor.

Interestingly, some components of the respiratory microbial flora have been linked to neurodegenerative and neuroinflammatory diseases: (1) *Chlamydia Pneumoniae* infection is associated with a 5-fold increased incidence of Alzheimer Disease (AD) (Maheshwari and Eslick, 2015) and *C. Pneumoniae* DNA was found in 90% of brain tissue samples in AD in comparison with 5% in controls (Balin et al., 1998); (2) Multiple Sclerosis (MS) has been linked to overrepresentation of nasal enterotoxin-A-producing *S. Aureus* (Brocke et al., 1993) and staphylococcal-specific oligoclonal bands (OCBs) are also identified in the cerebrospinal fluid of MS cases (Gay, 2013).

In conclusion, smell impairment is a widely represented and early pre-motor sign of PD (Ross et al., 2008; Doty, 2012). The olfactory system could be, together with gut, one of those peripheral sites where PD pathology first develops (Braak et al., 2003b; Hawkes et al., 2007). Unlike gut microbiota, consistent differences in nasal microbiota of PD patients have not been detected. Nevertheless, difficulties in sampling the area nearest to the olfactory bulb, together with high interindividual variability could be partially responsible for this result (Pereira et al., 2017; Heintz-Buschart et al., 2018).

## Taste

### Physiology

Taste is the body's other major chemosensory system and acts as the final arbiter of food acceptance or rejection behavior (Scott, 2005). Deficits in taste function has also been described in PD (Cossu et al., 2018; Cecchini et al., 2019).

There are five taste qualities (salty, sweet, bitter, sour, and umami. Recently, the ability to taste fatty acids has been described as a sixth taste quality Mattes, 2010. Sensory neurons project from taste buds to the gustatory nucleus (solitary nucleus) within the medulla oblongata via the cranial nerves VII, IX, and X. From here, neurons project to the thalamus ventral posteromedial nucleus to the primary gustatory cortical area (insular cortex and frontal operculum) (Maheswaran et al., 2014). Local projections from the solitary nucleus within the brainstem mediate unconscious responses, such as those related to eating behaviors (Tepper et al., 2014).

When patients report taste impairments it is difficult to attribute these to taste or smell abnormalities with certainty, given that during chewing and swallowing the two systems are both activated for the genesis of the taste sensation.

However, taste, individually, is only a reflection of perceptions originated from taste buds, i.e., sour, sweet, salty, bitter, savory (umami), and possibly chalky or metallic (Doty and Hawkes, 2019).

### Taste and PD

A few studies did an evaluation of taste in PD but the results have been not conclusive so far. The first report on the correlation between taste perception and PD is from 1993 (Travers et al., 1993). However, this study only investigated taste preferences, showing an increased preference for sucrose (sweet taste) in PD compared to healthy controls. Most of the studies on this topic published afterwards identified a reduced taste sensitivity in PD (ageusia) with an estimated frequency of taste impairment between 9 and 54% (Tarakad and Jankovic, 2017; Cossu et al., 2018; Doty and Hawkes, 2019). Only two groups showed higher sensitivity to taste stimuli in PD compared to controls (Sienkiewicz-Jarosz et al., 2013; Doty et al., 2015).

These contradictory results may be due to the relatively small sample and, most of all, may be due to the different measurement methods, which can be important potential confounders. For example, using small amounts of taste molecules through TST (Taste Strips test) seems more sensitive compared to the WMT (Whole Mouth Test), which investigates taste at a supra-threshold level. This has been reported in one study (Cecchini et al., 2014) where TST, but not WMT, showed a significant reduction in taste among PD patients compared to controls.

Moreover, Doty et al. and Sienkiewicz-Jarosz et al., using regional tests performed by placing the strip only on specific parts of the tongue, showed that relative intensity ratings had increased in the anterior tongue compared to the posterior tongue. These interesting results are not identified if the taste technique is limited to WMT. The authors speculated that PD-related damage to CN IX may release central inhibition on CN VII at the level of the brainstem, resulting in the observed enhancement of taste intensity on the anterior tongue (Sienkiewicz-Jarosz et al., 2013; Doty et al., 2015).

Finally, in addition to the techniques based on administration of chemicals on the tongue, electrogustometry (EGM) can be performed in taste research. The latter has been employed as a confirmative technique in a few studies on PD (Cecchini et al., 2014; Doty et al., 2015).

Furthermore, some studies on taste perception in PD showed a certain selectivity in its altered functions, interestingly identifying a specific reduced perceptivity for salty and bitter stimuli not reported for sweet and sour (Moberg et al., 2007; Sienkiewicz-Jarosz et al., 2013; Cecchini et al., 2014; Doty et al., 2015; Cossu et al., 2018). This is strenghtened by the finding that taste-receptor-genes are dysregulated in PD, particularly those accounting for the bitterness perception (Cossu et al., 2018).

These conflicting reports suggest that evidence might be too scarce to draw definitive conclusions about the effective clinical relevance of taste impairment in PD. However, it is now evident that taste can be affected in PD although less frequently than smell.

In this respect, olfaction impairment is acknowledged as an early biomarker of the disease, while taste impairment is rarely found in studies enrolling patients in the early phase. Moreover, taste impairment in PD has been preferentially reported in older patients, in association with PD dementia, psychosis and MCI (Lang et al., 2006; Cecchini et al., 2019). Whereas, with respect to incidence of taste impairment in different PD subtypes, no conclusive data are reported (Mun et al., 2016).

No specific treatment for taste impairment in PD exists (Tarakad and Jankovic, 2017), although zinc supplementation has been employed in treating taste disorders in the general population (Yagi et al., 2013).

The main aspects associated with taste impairment in PD are summarized in Table 1.

### Role of Taste Receptors in the PD Pathological Process

In the last few years some authors have focused their studies on specific taste performances in PD, identifying an increase in the frequency of the non-tasters for bitterness compared to healthy controls (Moberg et al., 2007; Cossu et al., 2018). The ability to perceive the bitter taste has gained considerable attention because of its genetic substrate. In the family of receptors for bitter, TAS2R38, a member of the T2R receptors, has been extensively studied, since the allelic diversity of the gene is able to explain much of the individual variability in the perception of the bitter taste. In fact, polymorphisms of the gene give rise to variants of the receptor with different affinity for the stimulus. T2R bitter taste receptors are G-protein coupled receptors originally identified on the tongue. Human nasal and bronchial airways (as well as gut mucosa) express multiple T2Rs isoforms (T2R4, T2R14, T2R16, and T2R38) (Janssen et al., 2011; Calvo and Egan, 2015). These T2Rs recognize bacterial products and, when activated, stimulate a signaling cascade involving calcium-driven nitric oxide production increasing ciliary beating as well as directly killing bacteria (Carey and Lee, 2019).

A cross-talk between signaling from T2Rs and toll-like receptors (TLRs) has been proposed by several authors (Lee and Cohen, 2015; Caputi and Giron, 2018; Maina et al., 2018). TLRs are a major class of pattern recognition receptors (PRRs) expressed in airway and intestinal cells that are able to activate inflammation through the transcription factor NF-KB. TLRs are strongly implicated in innate immunity by identifying conserved patterns principally found in microorganisms and their dysregulated signaling may play a role in α-synucleinopathy (Kim et al., 2018; Kwon et al., 2019).

Future studies will have to indicate whether the altered T2R observed in PD (Garcia-Esparcia et al., 2013; Lu et al., 2017; Cossu et al., 2018) may play a specific role in the inflammatory mechanisms associated with the initiation of misfolding of α-synuclein cascade possibly by modulating the innate immunity via TLR/T2R signaling.

## Conclusion

Our review confirms the relevance of smell and taste in the parkinsonian phenotype. Both hyposmia and hypogeusia are non-motor manifestations of PD that may impair quality of life.

Olfaction and taste impairment in PD are linked to different anatomical pathways. While alfa-synuclein pathology involves the olfactory tract and the anterior olfactory nucleus early in the disease course and smell impairment is often detectable years before the onset of motor symptoms of PD, the pathology at the basis of ageusia is less well-understood and the solitary tract is usually not involved by Lewy body pathology even in latest stages of the disease (Braak et al., 2003a). Nevertheless, typical PD pathological findings can be found in the operculum / anterior insular region, which is an important relay point for axons connecting to the orbitofrontal cortex (Braak and Del Tredici, 2009; Doty and Hawkes, 2019). Consequently, the topographical significance of taste impairment in PD most likely relies on the involvement of the cortex in the neurodegenerative process. Therefore, taste dysfunction can start early in the course of the disease but more frequently appears in advanced stages, in parallel with the advent of MCI (Braak stage 5), possibly reflecting a cognitive impairment (Cecchini et al., 2019).

These “chemosensory” symptoms are subject to ongoing research particularly focused on the relationship between enteric microbiota-derived factors and TLR/T2Rs engagement. The consequent signaling deriving from the receptor-activation in both the ENS and the CNS will provide novel insights into the complex dialogue between the host and the microbiota in PD. This research will perhaps, lead to prompt measures aimed at shaping the gut microbiota, aimed at ameliorate the function of intestinal epithelial barrier and restore a balance in the innate immune response in PD, in order to intercept and try to interfere with the early stages of the following neurodegenerative process.

## Author Contributions

GC, VO, IT, and MaM contributed conception and design of the study. VO and MaM wrote the first draft of the manuscript. MeM and IT wrote sections of the manuscript. All authors contributed to manuscript revision, read, and approved the submitted version.

### Conflict of Interest

The authors declare that the research was conducted in the absence of any commercial or financial relationships that could be construed as a potential conflict of interest.
